# Physiological Post-placental Cord Clamping in Pregnancies ≥34 Weeks Delivered by Cesarean Section: Maternal and Neonatal Outcomes From a Randomized Controlled Trial

**DOI:** 10.7759/cureus.94892

**Published:** 2025-10-18

**Authors:** Jignesh S Bulsara, Yogendra Waratkar, Devang Vadhu, Pratima Mehta, Arun K Singh, Manoja K Das

**Affiliations:** 1 Department of Obstetrics and Gynecology, Shrimad Rajchandra Hospital &amp; Research Centre, Dharampur, IND; 2 Department of Pediatrics, Shrimad Rajchandra Hospital &amp; Research Centre, Dharampur, IND; 3 Department of Hospital Administration, Shrimad Rajchandra Hospital &amp; Research Centre, Dharampur, IND; 4 Department of Pediatrics, Dr BC Roy Postgraduate Institute of Paediatric Sciences, Kolkata, IND; 5 Department of Neonatology, Institute of Health and Family Welfare, Kolkata, IND; 6 Department of Public Health, The INCLEN Trust International, New Delhi, IND

**Keywords:** cesarean section, early cord clamping, heart rate, neonatal hemodynamics, postpartum hemorrhage, post-placental separation cord clamping, pre-ductal oxygen saturation

## Abstract

Background

Cord clamping practices at birth vary widely. While early cord clamping (ECC) is the practice in cesarean sections (CS), post-placental separation cord clamping (PCC) is avoided due to the potential risk of maternal hemorrhage. This study aimed to document neonatal and maternal safety with PCC practice in CS delivery.

Methods

A prospective randomized controlled trial conducted between April and December 2022 included 211 pregnant women (PCC arm, n=102; ECC arm, n=109) with a gestation age of >34 weeks scheduled for CS delivery. In the PCC arm, the cord was clamped after placenta separation, and in the ECC arm, it was clamped after 30 seconds along with all other standard care.

The primary neonatal outcomes were the clinical outcomes with peripheral oxygen saturation (SpO_2_) and heart rate (HR) during the first 15 minutes of life. For the mothers, bleeding amount, postpartum hemorrhage, transfusion need, and hemoglobin change were documented.

Results

The neonatal parameters, including the SpO_2_ and HR values during the first 15 minutes, were comparable between the two groups, with no additional risk of adverse clinical outcomes.

Estimated maternal blood loss (402 mL (IQR 330-520 mL) vs. 350 mL (IQR 240-490 mL), p=0.05), mean hemoglobin change (0.8 gm/dL (0.3, 1.6 gm/dL) vs. 0.8 gm/dL (IQR 0.4, 1.5)) and blood transfusion need (6.8% vs. 10.0%) were comparable between the PCC and ECC groups, respectively.

Conclusions

Implementation of PCC during CS deliveries is feasible, potentially beneficial for term and late preterm neonates and safe for the mothers without any increased risk of bleeding.

## Introduction

The transition from intrauterine to extrauterine life at birth is a complex and coordinated process between the circulatory and respiratory systems. Usually, this transition is smooth; however, around 10% of newborns require some assistance, and 1% need extensive resuscitation support to initiate breathing [1]. The shift in infants’ respiratory function from placenta to lungs, coupled with the cardiovascular equilibrium, is smoother when the umbilical cord remains intact. Umbilical cord clamping before spontaneous breathing onset leads to loss of preload to the left ventricle and a drop in cardiac output, imitating a physiological shock situation [2]. Although these transitions occur very rapidly within the initial few minutes, reaching the point of circulatory equilibrium in the transition from placental to pulmonary respiration requires a variable amount of time, depending on the individual newborn and placental condition at birth [3].

If spontaneous respiration establishment is delayed, hypoxemia ensues, which may result in short-term and long-term consequences in the child. Only when pulmonary vascular resistance falls does pulmonary blood flow increase through right ventricular outflow and with left-to-right shunting through the ductus arteriosus. It has been reported that for every 10-second delay in cord clamping after spontaneous respiration up to two minutes, the risk of death or admission for newborns decreased by 20% among both normal-weight and low-birth-weight infants [4]. In a study among the very-low-birth-weight (VLBW) infants with delayed cord clamping (DCC), less delivery room resuscitation was needed than their counterparts with immediate cord clamping (ICC) (61% vs. 79%, p = 0.01) [5, 6].

Clinicians use heart rate (HR) and peripheral oxygen saturation (SpO_2_) primarily to guide neonatal resuscitation interventions. The currently used normal range of SpO_2_ in term and preterm neonates during the first 10 minutes of life was documented with early cord clamping (ECC) [5]. DCC after vaginal delivery has been shown to have higher SpO_2_ during the initial five minutes after birth than with ECC/ICC [7, 8]. Studies have shown that DCC has many immediate, short-term, and long-term benefits in infants, especially in preterm ones, including improved cardiopulmonary adaptation, reduced need for blood transfusions, and decreased incidence of intraventricular hemorrhage and necrotizing enterocolitis [9, 10].

The benefit of DCC is likely due to increased placental transfusion resulting in greater blood volume and initiation of ventilation before cord clamping [3, 11, 12]. As observed with ICC, there is a sudden drop in cardiac output, HR, and blood pressure, which is less often observed with DCC [13, 14].

The studies that compared DCC versus ECC have included newborns delivered vaginally only. During 2019-21, 21.5% of the Indian women delivered by cesarean section (CS), which has almost doubled over the last decade, with inter-state variations ranging from 5.2% to 60.7% [15]. The cardiopulmonary transition at birth for newborns delivered by CS mode differs from those delivered vaginally, which is also influenced by the maternal factors (antenatal and perinatal). The mechanism and rapidity of liquid absorption in the trachea and pulmonary airways may vary with the mode of delivery (vaginally or abdominally). The studies that intervened with ventilation first usually excluded the newborns delivered by CS. With a sizable proportion of newborns being delivered by CS, there is a need for documentation of the cardiopulmonary transition and trends of SpO_2_ and HR in newborns during the initial minutes after birth. The benefit of physiological post-placental separation cord clamping (PCC) has been documented among vaginally delivered term and preterm newborns [4, 5, 6, 7]. PCC appears to be a more physiological approach for the newborns delivered by CS, given the risk factors, variations in the time for cardiorespiratory transition, and thereby the tissue oxygenation and perfusion. But, there are concerns regarding the potential increased risk of maternal blood loss and postpartum hemorrhage with a delay in placental separation and suturing of the uterine incision. Additionally, there may be challenges regarding handling the baby with an intact cord. The studies that compared the effect of DCC vs. ECC with CS delivery have clamped the cord after 60 seconds. Thus, the maternal bleeding risk and outcomes with PCC need to be documented along with the newborn outcomes. 

We hypothesized that by adopting PCC, the ventricular preload and cardiac output would increase, which in turn would improve the SpO2 and minimize HR fluctuation. We also hypothesized that with PCC for CS delivery, there is no increased risk of maternal blood loss and adverse clinical outcomes in the mother and newborns during hospital stay compared to the ECC. We planned a randomized controlled trial to assess the effects of PCC after placental separation versus ECC (after 30 seconds) on pre-ductal SpO_2_ and HR up to 15 minutes after birth of term and late preterm newborns delivered by CS and maternal blood loss, and clinical outcomes. 

## Materials and methods

Study design and site

This open-label randomized controlled study was performed at Shrimad Rajchandra Hospital & Research Centre, a multispecialty mission hospital at Dharmapur, Valsad district, Gujarat, India, over six months (April-September 2022). The hospital has been practicing PCC for vaginal deliveries since 2018.

Participant selection

The study’s participants were 211 pregnant women who underwent CS delivery between April and December 2022. They were counselled and invited to participate in this study. Pregnant women aged >18 years, with ultrasound-documented singleton pregnancy, gestation age ≥34 weeks, and planned for CS delivery were eligible. The exclusion criteria were pregnancies with severe medical complications, fetal anomaly, fetal growth restriction, placental abruption, placenta previa, uterine rupture, fetal distress, intrauterine death, and Rh incompatibility. The infants born with signs of birth asphyxia, requiring urgent medical care, supplemental oxygen or ventilation, and/or medications at birth, major malformation, and stillbirths were also excluded. Written informed consent was obtained after explaining the study to the participants using participant information sheets in local languages. One of the obstetrician authors assessed the participants for eligibility and obtained consent. The indication for CS delivery was according to the hospital’s protocol.

Randomization

All participants were randomly allocated to one of the two study groups. Block randomization was performed by variable blocks of four and six using computer-generated numbers and sealed opaque envelopes. These envelopes were kept in the custody of an independent person, who opened the envelopes and informed the obstetrician, pediatrician, and nurses about the group allocation. Neither the doctors/nurses nor the patients were blinded. The laboratory team and data analysis team were unaware of the group allocation.

Procedure and study conduct

Before implementation, the nurses in the maternity operation theatre (OT) were trained on PCC, including handling the customized newborn care and resuscitation table and related newborn and maternal care. The obstetricians have been conducting vaginal delivery with PCC since 2018. Randomization for umbilical cord clamping timing (PCC or ECC) was done after recruitment of the pregnant woman, when the decision for CS was made. The CS was conducted under spinal anesthesia, which was performed by an anesthesiologist in the OT. All patients received standard care for transverse CS in the OT and recovery room and were shifted to the postpartum ward after two to three hours post-operation, once the doctor considered them stable. Immediately after delivery of the infant, a timer was turned on, and the umbilical cord was clamped according to the randomization group.

In the ECC group, the baby was placed on the mother’s chest till cord clamping (after 30 seconds). After cord clamping and cutting, the baby was handed over to the pediatrician, and intravenous oxytocin was administered. The placenta was removed manually.

In the PCC group, the cord was clamped after placental separation, and during the waiting time, the infant was placed on the mother’s chest for skin-to-skin contact and put to the nipple. The placenta was allowed to separate/deliver spontaneously. The placental separation was confirmed primarily by visual and palpation methods. The operating obstetrician noted the features of placental separation (lengthening of the umbilical cord and gush of blood), and the uterine changes were verified by palpation. If the placenta was not separated/delivered by 15 minutes or any increased bleeding was observed, manual separation was attempted. After placental separation, the cord was clamped and cut, and intravenous oxytocin was administered. As the placental separation takes several minutes, to enable essential newborn care at birth with an intact cord, a custom-designed resuscitation table is necessary. Our group designed a customized newborn care and resuscitation table, which was used for the CS deliveries with PCC. The pediatrician handled the infant and provided essential care according to the resuscitation guidelines. Breastfeeding was initiated for all newborns at the earliest. For all the infants, a pulse oximeter with a neonatal sensor (Radical-7, Masimo Corporation, Irvine, CA) was applied to the right palm or wrist of the newborn at the earliest after delivery to document the SpO_2_ and HR. The pulse oximeter was set to detect a signal every two seconds with maximal sensitivity. For the measurement of maternal bleeding, pre-weighed pads were used. The activities performed for the women and their newborns are summarized in Appendix A. 

Outcome measures

The primary outcomes were the SpO_2_ and HR transition during the first 15 minutes of the infant’s life. For the infants, the need for resuscitation, neonatal intensive care unit (NICU) care, phototherapy for jaundice, feeding practices (breastfeeding, top feeding, and galactagogue to mother), and the period of hospital stay were recorded. For the mothers, changes in hemoglobin (between admission and six to eight hours after operation), bleeding amount (vaginal and operation site), postpartum hemorrhage, blood transfusion, fluid infusion, and vitals during and after surgery were documented. Maternal bleeding was documented by the gravimetric method (using preweighed pads/sponges and measuring the suction containers). The hemoglobin was analyzed by an automated hematology analyzer.

Data collection

Information on maternal (age, weight, height, parity, pregnancy history, hemoglobin, vitals, blood loss, and hospital care) and neonatal parameters (sex, weight, height, head and chest circumference, Apgar (Appearance, Pulse, Grimace, Activity, and Respiration) scores at one and five minutes, hospital care) was obtained from the clinical records. The pre-ductal SpO_2_ and HR values recorded by the pulse oximeter were extracted in Microsoft Excel format (Microsoft Corp., Redmond, WA) using Trendcom software (Masimo Corporation).

Data analysis

Data points with missing data and alarm messages (sensor off, no sensor, defective sensor, low perfusion, and low signal) were excluded. The intention-to-treat analysis approach was followed for statistical analysis. Descriptive analysis was done with numbers and proportions for categorical variables, means and standard deviations for normally distributed continuous variables, or medians and IQRs for variables with skewed distribution. The values between groups were compared for statistical significance using Student’s t-test or Mann-Whitney U test and Fisher's exact test, depending on the skewness. The groups were compared using a linear regression analysis for continuous variables. Logistic regression analysis was used for categorical variables. All tests were two-sided, and a p-value < 0.05 was considered statistically significant. Statistical analysis was performed using STATA version 15.0 (StataCorp LLC, College Station, TX). The sample size was estimated considering an anticipated difference in the HR between DCC (mean 145, SD 20) and ECC (mean 155, SD 22) groups during the first five minutes, with 80% power and 95% confidence level (two-sided), which was 70 participants per group. This sample size would be adequate to detect a significant difference in the maternal blood loss between the DCC (mean 400 ml, SD 30 ml) and ECC (mean 350 ml, SD 30 ml) groups, with 80% power and 95% confidence level (two-sided). 

Ethical considerations

Informed written consent was obtained from the eligible women before delivery. Confidentiality in data handling was maintained. The study protocol was reviewed and approved by the Institute Ethics Committee (reference number: HEC/01/SRHEC-2019).

Trial registration

The trial was registered in the Clinical Trial Registry-India (registration number: CTRI/2022/03/041025).

## Results

**Figure 1:**
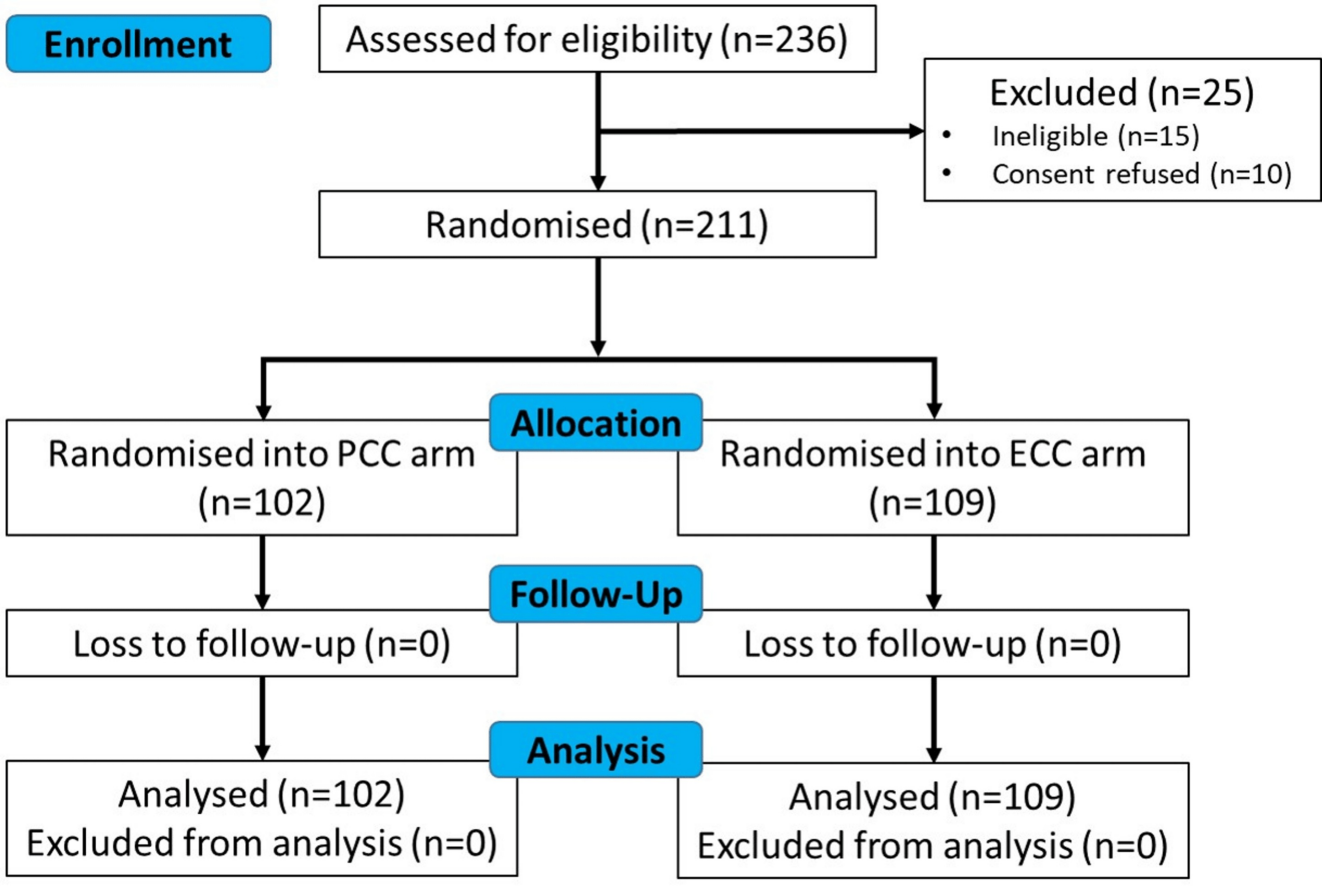
A CONSORT diagram showing participant recruitment and allocation in the randomized trial PCC: physiological cord clamping; ECC: early cord clamping; CONSORT: Consolidated Standards of Reporting Trials

Out of 236 pregnant women who underwent CS, 221 were found eligible, and 211 consented. Based on randomization, 102 women received PCC and 109 received ECC (after 30 seconds) (Figure 1).

Maternal characteristics are shown in Table 1. The participants in the two groups were similar for all the parameters except the parity status; the PCC group had more multiparous mothers.

**Table 1:** Profile of the study participants (delivered mothers) P-values were calculated using the Mann-Whitney U test for continuous variables, the chi-square test for categorical variables, and Fisher’s exact test when cell frequency was ≤5; p < 0.05 was considered significant. Value indicates the z value for Mann Mann-Whitney U test and the chi-square test value. PCC: physiological cord clamping; ECC: early cord clamping

Parameters	PCC (n=102)	ECC (n=109)	P-value	Test	Value
Age					
Age in years, median (IQR)	24 (22,27)	24 (21,27)	0.75	Mann-Whitney	0.31
<25 years, n (%)	60 (58.82)	62 (56.88)	0.793	Fisher’s exact	
25-35 years, n (%)	39 (38.24)	45 (41.28)			
>35 years, n (%)	3 (2.94)	2 (1.83)			
Gestational age					
Gestational age (weeks), median (IQR)	40 (40,40)	40 (40,40)	0.75	Mann-Whitney	0.32
≤37week, n (%)	12 (11.76)	19 (17.43)	0.245	Chi-square	1.35
>37week, n (%)	90 (88.24)	90 (82.57)			
Gravida					
Gravida, median (IQR)	2 (1,2)	2 (1,2)	0.58	Mann-Whitney	0.55
Primigravida, n (%)	33 (32.35)	47 (43.12)	0.20	Fisher’s exact	
2^nd ^- 3^rd^ gravida, n (%)	67 (65.69)	59 (54.13)			
≥4^th^ gravida, n (%)	2 (1.96)	3 (2.75)			
Parity					
Parity, median (IQR)	1 (0,1)	1 (0,1)	0.41	Mann-Whitney	0.82
Primiparous, n (%)	34 (33.33)	48 (44.04)	0.11	Chi-square	2.54
Multiparous, n (%)	68 (66.67)	61 (55.96)			
Education					
≤5 standard, n (%)	14 (13.73)	9 (8.26)	0.40	Chi-square	1.82
6-10 standard, n (%)	42 (41.18)	51 (46.79)			
>10 standard, n (%)	46 (45.10)	49 (44.95)			
Indication					
Emergency, n (%)	81 (79.41)	94 (86.24)	0.19	Chi-square	1.74
Elective, n (%)	21 (20.59)	15 (13.76)			

The clinical parameters at admission, after CS, and outcomes for the mothers are presented in Table 2. The median placenta separation time was 208 seconds (IQR 130-310 seconds) and 36 seconds (IQR 30-44 seconds) in the PCC and ECC groups, respectively. The median cord clamping and median oxytocin administration time were 227.5 seconds (IQR 135-330 seconds) and 45 seconds (IQR 36-55 seconds) and 231.5 seconds (IQR 165-320 seconds), and 60 seconds (IQR 47-80 seconds), respectively. The maternal hemoglobin category of anemia at admission and after CS was comparable. There was a rise in the proportion of mothers with moderate anemia after the delivery, which was similar in both groups. The mean maternal hemoglobin change was similar in the two groups. Although the median total bleeding amount was higher by 52 ml in the PCC group (PCC: 402.5 ml vs. ECC: 350 ml), no woman in any group had PPH. Eleven women (10.09%) in the ECC group needed blood transfusions compared to seven (6.86%) in the PCC group, and the difference was not statistically significant. None of the women required an additional oxytocin dose for control of PPH.

**Table 2:** Maternal clinical outcomes P-values were calculated using the Mann-Whitney U test for continuous variables, chi-square test for categorical variables, and Fisher’s exact test when cell frequency ≤5. Values represent the z statistic for Mann-Whitney U and Pearson’s chi-square statistic. A p-value <0.05 was considered significant. IQR: interquartile range; CS: cesarean section; PCC: physiological cord clamping; ECC: early cord clamping; bpm: beats per minute

Parameters	PCC (n=102)	ECC (n=109)	p-value	Test	p-value
Hemoglobin at admission					
Hemoglobin at admission (gm/dL), median (IQR)	10.8 (10, 11.7)	10.8 (10, 11.7)	0.78	Mann-Whitney	0.27
No anemia (≥11 gm/dL), n (%)	44 (43.14)	45 (41.28)	0.88	Chi-square	0.27
Mild anemia (10-10.9 gm/dL), n (%)	34 (33.33)	40 (36.70)			
Moderate anemia (7-9.9 gm/dL) (n (%)	24 (23.53)	24 (22.02)			
Hemoglobin after CS					
Hemoglobin after CS (gm/dL), median (IQR)	10 (9, 10.7)	10 (9.1, 10.6)	0.96	Mann-Whitney	-0.052
No anemia (≥11 gm/dL), n (%)	16 (15.69)	15 (13.76)	0.92	Chi-square	0.16
Mild anemia (10-10.9 gm/dL), n (%)	36 (35.29)	40 (36.70)			
Moderate anemia (7-9.9 gm/dL), n (%)	50 (49.02)	54 (49.54)			
Hemoglobin change					
Hemoglobin change (gm/dL), median (IQR)	0.8 (0.3, 1.6)	0.8 (0.4, 1.5)	0.93	MannWhitney	-0.084
Blood transfusion needed, n (%)	7 (6.86)	11 (10.09)	0.40	Chi-square	0.70
Amount of bleeding					
Total bleeding (ml), median (IQR)	402.5 (330, 520)	350 (240, 490)	0.05	Mann-Whitney	1.921
Operation site bleeding (ml), median (IQR)	365 (300, 470)	320 (200, 450)	0.07	Mann-Whitney	1.81
Vaginal bleeding (ml), median (IQR)	40 (30, 50)	40 (30, 50)	0.64	Mann-Whitney	0.47
Postpartum hemorrhage, n (%)	0 (0)	0 (0)	-		
Cord clamping and placenta handling					
Cord clamping time (seconds), median (IQR)	227.5 (135,330)	45 (36,55)	<0.05	Mann-Whitney	12.12
Cord clamped <30 sec, n (%)	0 (0)	8 (7.34)	<0.05	Fisher’s exact	
Cord clamped 31 sec- 180 sec, n (%)	40 (39.22)	101 (92.66)			
Cord clamped >180 sec, n (%)	62 (60.78)	0 (0)			
Placenta separation time (sec), median (IQR)	208 (130, 310)	36 (30, 44)	<0.05	Mann-Whitney	12.37
Oxytocin administration time (sec), median (IQR)	231.5 (165,320)	60 (47,80)	<0.05	Mann-Whitney	11.43
Duration of hospital stay (days), median (IQR)	4 (3,4)	4 (3,5)	0.78	Mann-Whitney	-0.27

The maternal status of vitals (pulse rate, blood pressure, and SpO_2_) before, during, and after surgery is given in Table 3.

**Table 3:** Maternal vitals before, during and after surgery P-values were calculated using the Mann-Whitney U test for continuous variables, chi-square test for categorical variables, and Fisher’s exact test when cell frequency ≤5. Values represent the z statistic for the Mann-Whitney U and Pearson's chi-square statistic. A p-value <0.05 was considered significant. PCC: physiological cord clamping; ECC: early cord clamping; HR: heart rate; bpm: beats per minute

Parameters	PCC (n=102) n (%)	ECC (n=109) n (%)	p-value	Test	Value
On admission					
Maternal tachycardia (HR >100 bpm)	1 (0.98)	4 (3.67)	0.37	Fisher’s exact	
SpO_2_ <94%	2 (1.96)	1 (0.92)	0.61	Fisher’s exact	
Systolic blood pressure >140 mmHg	10 (9.80)	11 (10.09)	0.94	Chi-square	0.005
Diastolic blood pressure >90 mmHg	7 (6.86)	13 (11.93)	0.21	Chi-square	1.57
Fetal tachycardia (FHR >160 bpm)	0 (0)	1 (0.92)	1	Fisher’s exact	
Before surgery					
Maternal tachycardia (HR >100 bpm)	15 (14.71)	27 (24.77)	0.07	Chi-square	3.35
SpO_2_ <94%	2 (1.96)	0 (0)	0.23	Fisher’s exact	
Systolic blood pressure >140 mmHg	6 (5.88)	4 (3.67)	0.52	Fisher’s exact	
Diastolic blood Pressure >90 mmHg	7 (6.86)	6 (5.50)	0.68	Chi-square	0.17
Maternal tachycardia (HR >100 bpm)	1 (0.98)	1 (0.92)	1	Fisher’s exact	
During surgery					
Maternal tachycardia (HR >100 bpm)	11 (10.78)	19 (17.43)	0.17	Chi-square	1.91
SpO_2_ <94%	2 (1.96)	1 (0.92)	0.61	Fisher’s exact	
Systolic blood pressure >140 mmHg	2 (0.98)	3 (2.75)	0.62	Fisher’s exact	
Diastolic blood Pressure >90 mmHg	4 (3.92)	5 (4.59)	1	Fisher’s exact	
Immediate post-surgery					
Maternal tachycardia (HR >100 bpm)	6 (5.88)	4 (3.67)	0.52	Fisher’s exact	
SpO_2_ <94%	0 (0)	2 (1.83)	0.49	Fisher’s exact	
Systolic blood pressure >140 mmHg	3 (2.94)	4 (3.67)	1	Fisher’s exact	
Diastolic blood Pressure >90 mmHg	5 (4.90)	4 (3.67)	0.74	Fisher’s exact	
6 hours after surgery					
Maternal tachycardia (HR >100 bpm)	0 (0)	1 (0.92)	1	Fisher’s exact	
SpO_2_ <94%	1 (0.98)	4 (3.67)	0.37	Fisher’s exact	
Systolic blood pressure >140 mmHg	3 (2.94)	1 (0.92)	0.35	Fisher’s exact	
Diastolic blood Pressure >90 mmHg	3 (2.94)	3 (2.75)	1	Fisher’s exact	

Regression analysis did not find the maternal parameters to have a significant influence on the amount of bleeding. 

The neonatal parameters at birth and during hospital stay are presented in Table 4. The neonatal characteristics were similar in both groups, such as gestational age, birth weight, Apgar scores at one and five minutes, resuscitation need, admission to NICU, jaundice requiring phototherapy, breastfeeding, and the period of hospital stay. Two neonates in each group required resuscitation with positive pressure ventilation. The two neonates in the PCC group were given resuscitation with an intact cord. There was no difference in the proportion of neonates admitted to the neonatal ICU or special care unit and requiring phototherapy between the groups. The breastfeeding and the need for top feeding were similar.

**Table 4:** Neonatal clinical outcomes P-values were calculated using the Mann-Whitney U test for continuous variables, chi-square test for categorical variables, and Fisher’s exact test when cell frequency ≤5. Values indicate the z statistic for the Mann-Whitney U and the Pearson chi-square statistic. A p-value <0.05 was considered significant. PCC: physiological cord clamping; ECC: early cord clamping; Apgar: appearance, pulse, grimace, activity, and respiration; hr: hour

Parameters	PCC (n=102)	ECC (n=109)	P-value	Test	p-value
Gestational age					
Full term (>37 weeks), n (%)	101 (99.02)	106 (97.25)	0.62	Fisher's exact	
Preterm (<37 weeks), n (%)	1 (0.98)	3 (2.75)			
Anthropometry					
Birthweight (gm), median (IQR)	2615 (2270, 2990)	2630 (2280, 2950)	0.72	Mann-Whitney	0.35
Birthweight <2500 gm, n (%)	39 (38.24)	42 (38.53)	0.97	Chi-square	0.002
Birthweight ≥ 2500 gm, n (%)	63 (61.76)	67 (61.47)			
Apgar score					
Apgar score, median (IQR)	8 (8, 8)	8 (8, 8)	0.78	Mann-Whitney	0.28
Apgar score range 4-6, n (%)	1 (0.98)	0	0.48	Fisher’s exact	
Apgar score range 7-10, n (%)	101 (99.02)	109 (100)			
Clinical care needs					
Admitted to the NICU, n (%)	21 (20.59)	19 (17.43)	0.56	Chi-square	0.34
Resuscitation needed, n (%)	2 (1.96)	2 (1.83)	0.9	Fisher’s exact	
Jaundice needed phototherapy, n (%)	2 (1.96)	3 (2.75)	1	Fisher’s exact	
Breastfeeding initiated <1 hr, n (%)	84(82.35)	94(86.24)	0.45	Chi-square	0.60
Needed top feeding, n (%)	2 (1.96)	3 (2.75)	1	Fisher’s exact	
Mother given galactogogue, n (%)	0	1 (0.92)	1	Fisher’s exact	
Period of hospital stay (days), median (IQR)	3 (3, 4)	3 (3, 5)	0.58	Mann-Whitney	-0.55

The hemodynamic parameters (HR and SpO_2_) transition pattern in the neonates is shown in Table 5. The neonates in the PCC group had higher (by 5%) SpO_2_ and lower (by 13 beats per minute (bpm)) HR values during the initial 30 seconds, and thereafter these values were closer. The time to reach a pre-ductal SPO_2_ level of 80%, 85%, 90% and 95% was shorter for the neonates in the PCC group than the ECC group. 

**Table 5:** The pattern of HR and SPO2 transition in the neonates during first 15 minutes of life bpm: beats per minute; ECC: early cord clamping; HR: heart rate; PCC: physiological post-placental separation cord clamping; SD: standard deviation; SPO_2_: pre-ductal arterial oxygen saturation

Parameters	PCC (n=102) Mean (SD)	ECC (n=109) Mean (SD)	p-value
Heart rate (bpm)			
30 seconds	137 (4.2)	150 (3.7)	<0.0001
60 seconds	156 (14.7)	158 (12.2)	0.27
90 seconds	152 (20.1)	157 (13.4)	0.03
120 seconds	156 (16.4)	155 (15.5)	0.64
180 seconds	145 (22.4)	155 (16.1)	0.0002
300 seconds	151 (18.8)	153 (15.0)	0.39
600 seconds	150 (13.6)	150 (14.0)	1.0
900 seconds	144 (10.6)	145 (14.1)	0.56
Pre-ductal SpO_2_ (%)			
30 seconds	80 (8.6)	75 (7.6)	<0.0001
60 seconds	80 (15.4)	80 (10.4)	1.0
90 seconds	83 (12.3)	82 (10.4)	0.52
120 seconds	84 (14.6)	85 (11.4)	0.57
180 seconds	85 (14.8)	85 (9.9)	1.0
300 seconds	86 (10.8)	88 (9.9)	0.16
600 seconds	90 (6.8)	91 (6.0)	0.25
900 seconds	95 (2.8)	95 (3.7)	1.0
Time to reach target SpO_2_ (min)			
Time to reach SpO_2_ 80%	3.09 (2.57)	3.59 (3.20)	0.21
Time to reach SpO_2_ 85%	3.35 (3.25)	4.37 (3.38)	0.02
Time to reach SpO_2_ ​​​​​​​90%	3.58 (3.17)	5.46 (4.00)	0.0002
Time to reach SpO_2_ ​​​​​​​95%	4.47 (3.33)	7.09 (4.56)	<0.0001

## Discussion

In this RCT, higher SpO_2_ levels with lower HR and a lower fluctuating trend throughout the first 15 minutes of life in the neonates with PCC practice reflected a smoother transition after birth compared to the neonates with ECC practice. There was no significant difference in the clinical outcomes for the neonates, including the risk of admission to the NICU or phototherapy need. With the adoption of PCC practice for CS delivery with ≥34+0 weeks of gestation, no increased risk of blood loss, PPH, anemia, need for additional oxytocin, or blood transfusion in mothers was observed. These findings support that PCC can be safely used for CS deliveries without any increased risk for the mothers and neonates. It further indicates the benefit of PCC practice for the neonate with better hemodynamic transition.

We could retrieve one study that compared the effect of physiologically based cord clamping (PBCC) vs. ECC with CS deliveries with gestation ≥32 weeks (n=163; PBCC, n=63; ECC, n=60). The cord was clamped at a mean of 136 seconds and 37 seconds in the PBCC and ECC groups, respectively. The maternal blood losses in the PBCC and ECC groups were 469 ml (SD 326) and 518 ml (SD 573), respectively, and maternal PPH was similar between the two groups (PPH >500 ml, PBCC- 24% vs ECC 23% and PPH >1000 ml, PBCC- 10% vs ECC 8%) [16]. 

In a study in Argentina using cord clamping at 15 seconds, 60 seconds, and 180 seconds (including both vaginal and abdominal deliveries), no significant difference was observed in the maternal blood loss volume (median and IQR, 265 mL, 250 mL, and 300 mL, respectively), PPH (blood loss >500 mL 26.8%, 22.2%, and 25.4%, respectively; blood loss >1000 mL 3.6%, 5.6%, and 3.2%, respectively), and maternal hematocrit 24 hours after birth (29.9%, SD 3.5; 30.9%, SD 4.5; and 30.6%, SD 3.6). No segregated data for the vaginal and abdominal deliveries were presented in the study [17].

A study in Thailand documented no significant difference in the intraoperative maternal blood loss with adoption of cord clamping at 30 seconds (274.7 ml) and 60 seconds (272.5 ml) in term pregnancies with elective CS [18]. Another study in Thailand comparing ECC (<30 seconds) and DCC (>30 seconds) observed no significant difference in the maternal blood loss (ECC 848.4 ± 374.3 ml and DCC 848.5 ± 255.1 ml) and mean hemoglobin decline (ECC 2.0 ± 1.5 g/dL and DCC 2.1 ± 1.6 g/dL) [19].

A systematic review (two studies) observed that adoption of different cord clamping timings with CS for term pregnancies had no significant difference in the maternal bleeding and PPH risk [20]. One of these studies among American pregnant women who underwent elective CS with cord clamping at 90-120 seconds documented lesser intraoperative blood loss (174 mL, 95% CI -61, − 286), with blood loss of 691 ml (SD 218) in the DCC (90-120 sec group) and 864 ml (SD 442) in the ECC group (historical control). There were also significantly fewer blood transfusions needed for the mothers in the DCC group (2.7%) compared to the ECC group (18.8%) [21].

Another study among Sri Lankan women with term pregnancies delivered by CS with cord clamping at < 15 seconds, 60-75 seconds, or 120-135 seconds had comparable operative blood loss between the groups (operative blood loss in ml: 492 (95% CI 462-522), 523 (95% CI 488-558), and 493 (95% CI 455-531)) and was statistically not significant [22].

A study from New York observed that cord clamping at 15 seconds (ECC) or 60 seconds (DCC) after delivery for CS at term gestation did not show a significant difference in the median operative blood loss (ECC 800 ml, IQR 800-1000 ml; DCC 800 ml, IQR 800-800 ml), and PPH >1000 ml blood loss (ECC 8.8%; DCC 7.1%) [23]. Another study from the United States observed no difference in maternal blood loss between ECC (median 800 ml, IQR 7000-1000 ml) and DCC (>30 seconds, median 800 ml, IQR 7000-1000 ml) with CS delivered at term and had lower PPH with DCC [24].

A study in the Netherlands compared DCC (after 180 seconds) with ECC (<15 seconds) for the CS in term pregnancies and found no statistically significant difference in the maternal operative bleeding (median 400 ml, IQR 300-600 ml) [25]. A study among Italian pregnant women who delivered at term observed that DCC (after 180 seconds) had less maternal operative bleeding (mean 469 ml, SD 292 ml) compared to those with ECC (<15 seconds) (563 ml, SD 415 ml) [26]. A study among Chinese patients who underwent CS at term gestation documented lower maternal operative bleeding (156.8 ±87.4 ml) with DCC (after 180 seconds) compared to the ECC (221.6 ±197.2 ml) [27].

Pregnant women who delivered at term observed that DCC (after 180 seconds) had less maternal operative bleeding (mean 469 ml, SD 292 ml) compared to those with ECC (<15 seconds) (563 ml, SD 415 ml) [26]. 

A study from India using DCC (>120 seconds) for women delivering by CS at term observed no increased risk of maternal operative bleeding and PPH. Compared to ECC (<15 seconds), the newborn delivered with DCC had a higher birth weight (400 gms), a better Apgar score at one and five minutes, with comparable phototherapy need [28].

In an earlier study by our group comparing PCC vs. ECC for term and late preterm (>34 weeks of gestation) neonates born vaginally, we documented a smoother transition with HR and SpO_2 _during the initial five minutes after birth with PCC. In the current study, we observed higher HR for the newborns born by CS in both the groups (PCC and ECC) during the initial three minutes compared to the newborns born vaginally, as observed in the previous study [7]. 

The neonatal resuscitation guidelines generally refer to Dawson’s nomogram for the preductal SpO₂ >60 to 65% by two minutes and >80% by 5 minutes as the lower acceptable levels [29, 30]. Our study findings indicate that with PCC practice, the neonates achieve these target SpO2 levels earlier.

The post-hoc power calculations for the difference observed in HR at 180 seconds were 96%, and for the total amount of maternal bleeding, it was 86%. The trial provided an opportunity to test the feasibility of using the customized newborn care and resuscitation table with PCC practice. Based on the user feedback, this table has been further refined to improve the ease of operation.

While the overall observations indicate no safety concerns for the mother with the use of PCC with CS delivery, and there are potential benefits for the newborn, some modifications in the procedures during the CS shall be needed for handling the baby with an intact cord during the waiting period for placental separation. But, this also provides an opportunity for practicing and promoting immediate skin-to-skin care and early initiation of breastfeeding in CS deliveries. 

Strengths and limitations

A systematic parallel randomized controlled trial approach with a head-on comparison between the ECC and PCC practices using a customized newborn care and resuscitation table is a strength. A combination of methods for documentation of maternal blood loss with CS, involving direct measurements and weighing, allowed accurate estimation. Despite careful effort by the operating obstetrician, there would have been some contamination of towels and drapes with amniotic fluid, which could have introduced a small margin of error. The imbalances in multiparous women in the two groups (more in the PCC group) could have affected the maternal outcome. We did not follow up with the infants to document the longer-term outcomes.

## Conclusions

To conclude, our results indicate that the PCC approach for CS deliveries is potentially beneficial for term and late preterm neonates, with better and smoother hemodynamic transition during the initial 15 minutes of life than the ECC practice, with no safety concern for the mother in terms of bleeding, risk of PPH, and anemia. It indicates that PCC is feasible and safe to practice in CS and also neonatal resuscitation, with a customized table. The additional opportunities for initiating immediate skin-to-skin contact practices could assist in preventing hypothermia and early breastfeeding initiation in babies with PCC practice. This mandates further investigation and clinical practice alongside documentation of the long-term outcome data in a larger and multicenter study population.

## Figures and Tables

**Figure 2:**
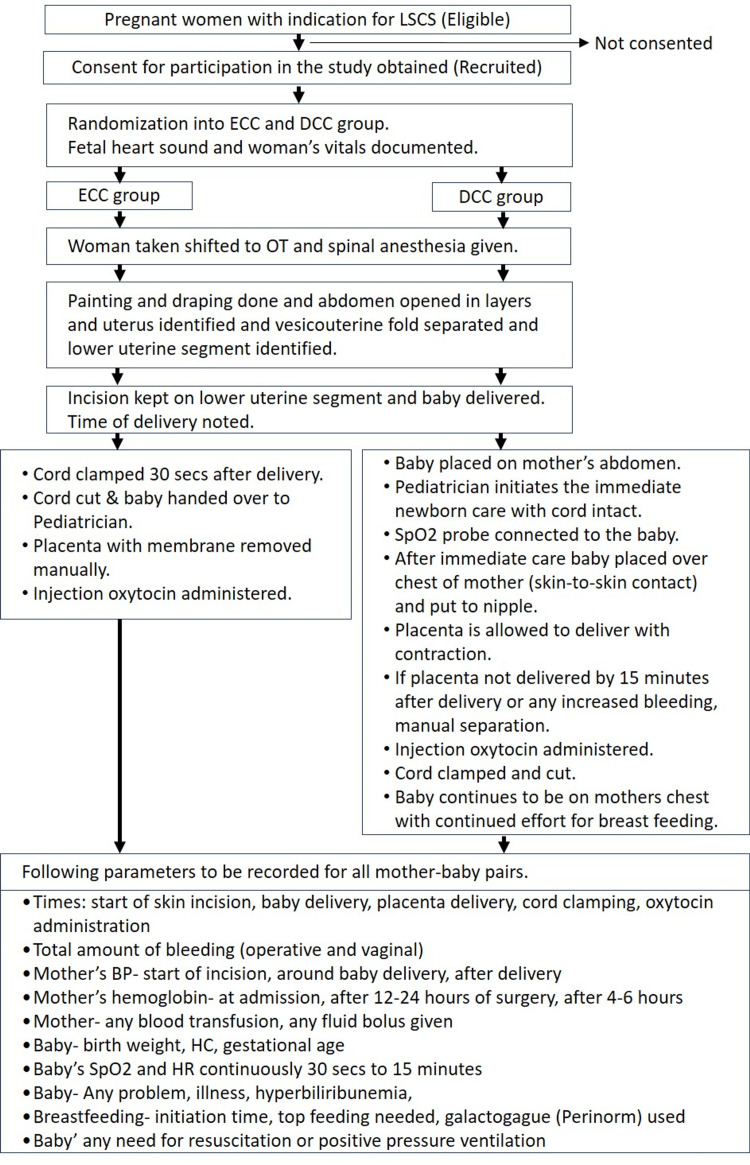
A flowchart of the activities undertaken for the pregnant mothers and neonates in the study LSCS: lower segment cesarean section; PCC: physiological cord clamping; ECC: early cord clamping

## Appendices

Appendix A 
